# Electroacupuncture alleviates neuropathic pain in a rat model of CCD via suppressing P2X3 expression in dorsal root ganglia

**DOI:** 10.1186/s13020-024-01030-9

**Published:** 2024-11-11

**Authors:** Yu Zheng, Minjian Jiang, Zhouyuan Wei, Hengyu Chi, Yurong Kang, Siyi Li, Yinmu Zheng, Xiaofen He, Xiaomei Shao, Jianqiao Fang, Yongliang Jiang

**Affiliations:** 1 Key Laboratory of Acupuncture and Neurology of Zhejiang Province, Department of Neurobiology and Acupuncture Research, The Third Clinical Medical College, Zhejiang Chinese Medical University, Hangzhou, Zhejiang China; 2 Zhejiang Chinese Medical University, Hangzhou, Zhejiang China

**Keywords:** EA, Chronic compression, DRG, Neuropathic pain, P2X3 receptor

## Abstract

**Background:**

Sciatica and low back pain are prevalent clinical types of neuropathic pain that significantly impair patients' quality of life. Conventional therapies often lack effectiveness, making these conditions challenging to treat. Electroacupuncture (EA) is an effective physiotherapy for pain relief. Prior research has demonstrated a relationship between the frequency of neuropathic pain and the analgesic impact of EA stimulation. This work aimed to assess the analgesic effects of EA in a rat model of chronic compression of the dorsal root ganglion (CCD) and to understand the underlying processes.

**Methods:**

We established a rat CCD model to simulate sciatica and low back pain. EA was applied to rats with CCD at various frequencies (2 Hz, 100 Hz, and 2/100 Hz). The paw withdrawal threshold (PWT) was measured to assess analgesic effects. Additionally, protein levels of the purinergic receptor P2X3 (P2X3) and the expression of nociceptive neuronal markers were analyzed using immunohistochemistry and western blot (WB) techniques. The study also measured levels of proinflammatory cytokines TNF-α and IL-1β in the dorsal root ganglion (DRG). The involvement of P2X3 receptors was further investigated using the P2X3 agonist, α,β-methylene ATP (α,β-meATP).

**Results:**

CCD rats developed pronounced mechanical allodynia. EA stimulation at all tested frequencies produced analgesic effects, with 2/100 Hz showing superior efficacy compared to 2 Hz and 100 Hz. The expression of P2X3 was increased in ipsilateral DRG of CCD model rats. P2X3 were co-labeled with isolectin B4 (IB4) and transient receptor potential vanilloid (TRPV1), indicating their role in nociception. 2/100 Hz EA treatment significantly reduced mechanical allodynia and inhibited the overexpression of P2X3, TRPV1, substance P (SP), and calcitonin gene-related peptide (CGRP) in the ipsilateral DRG of CCD model rats. Additionally, EA reduced the levels of proinflammatory cytokines TNF-α and IL-1β in the ipsilateral DRG, indicating an anti-inflammatory effect. The P2X3 agonist α,β-me ATP attenuated the analgesic effect of 2/100 Hz EA in CCD rats. The WB and immunofluorescence results consistently demonstrated P2X3 inhibition contributed to the analgesic effects of 2/100 Hz EA on CCD-induced neuropathic pain.

**Conclusions:**

Our findings suggest that 2/100 Hz EA alleviates neuropathic pain in rats by inhibiting the upregulation of P2X3 receptors in the ipsilateral DRG. This study backs up EA as a viable treatment option for sciatica and low back pain in clinical settings.

## Introduction

Neuropathic pain, including sciatica and low back pain, is a prevalent symptom in various clinical disorders that significantly impacts patients' quality of life [1]. Chronic compression of the dorsal root ganglion (DRG) or its surrounding nerve roots, as seen in conditions like spinal trauma, intervertebral disc herniation, or intervertebral foramen stenosis, contributes to these pain syndromes [2–5]. The CCD model in rats, mimicking these clinical conditions, has exhibited spontaneous pain, nociceptive hypersensitivity, and abnormal pain responses, making it a suitable model for studying radicular pain mechanisms [6, 7].

P2X3 receptors belong to the purinergic receptor family. They are crucial for sensory neurotransmission and peripheral pain processing [8]. ATP activates these ligand-gated ion channels, leading to depolarization, calcium influx, and the release of pro-inflammatory mediators, thus modulating neuronal excitability and pain perception [9–11]. P2X3 has been connected to pain responses in a number of animal models of pain. It is strongly expressed in tiny and medium-sized sensory neurons in DRGs, including the diabetic neuropathic pain (DNP) model, the bone cancer pain (BCP) model, the spared nerve injury (SNI) model, and the Fuchs' complete adjuvant (CFA) model [12–15]. Pharmacological targeting of P2X3 receptors has emerged as a promising therapeutic method for managing chronic pain disorders. Numerous selective antagonists demonstrating efficacy in preclinical models and clinical studies [10].

Studies have shown that P2X3 receptors are primarily expressed on Isolectin B4 (IB4)-positive class C fibers. The significance of P2X3 in nociceptive signaling and neuropathic pain is indicated by its colocalization with IB4-positive neurons [16]. Moreover, P2X3 and Transient Receptor Potential Vanilloid 1 (TRPV1) receptors have a significant relationship. Both contribute to pain transmission pathways and are co-expressed in sensory neurons. Increased intracellular calcium following P2X3 receptor activation may indirectly activate TRPV1 channels, intensifying nociceptive signals [17]. The production of neuropeptides like substance P (SP) and calcitonin gene-related peptide (CGRP) is likewise controlled by P2X3 receptors. P2X3 receptor activation can increase central sensitization and pain transmission by causing the primary sensory neurons to produce SP and CGRP [18, 19].

Acupuncture is currently one of the most important means of treating chronic pain, and electroacupuncture (EA) has demonstrated promising analgesic effects on radicular pain caused by nerve root compression [20]. Numerous studies have proved the usefulness of EA in reducing chronic pain, with different frequencies of stimulation producing varying analgesic effects and mechanisms [21]. Previous study found that DNP can be relieved by 2 Hz and 100 Hz EA stimulation. However, the analgesic effect of 2 Hz EA is stronger [22]. Some studies have concluded that both 2 Hz and 100 Hz EA can alleviate neurogenic pain, with 2 Hz EA demonstrating superior analgesic efficacy compared to 100 Hz [23]. Moreover, Xu et al. [24] found that 2/100 Hz EA is an optimal therapeutic scheme for Cancer-Induced Bone Pain. Recent studies have shown that EA at Zusanli and Kunlun can modulate pain through various mechanisms, such as reducing inflammatory cytokine levels, modulating pain-related neurotransmitters, and affecting pain signaling pathways [25]. There are differences in the mechanism of EA analgesia in different pain models. At present, the optimal frequency of EA for the treatment of CCD needs to be studied, and it is clinically significant to investigate the optimum frequency of EA for CCD. The exact mechanisms of EA’s analgesic effect on CCD are still not fully understood. Mechanistic studies of EA’s analgesic effect would help to validate its potential therapeutic effect on CCD.

In this project, we establish a continuous compression model of the DRG and screen the optimal EA treatment options with different frequencies on CCD model rats. We also explored the possible involvement of P2X3 in EA-mediated analgesia.

## Materials and methods

### Animals

Healthy male SD (Sprague Dawley) rats of SPF grade were purchased from the Shanghai Laboratory Animal Center of the Chinese Academy of Sciences (SCXK (Shanghai) 2022–0004), weighing 200–250 g, and housed in the Laboratory Animal Center of Zhejiang Chinese Medical University (SYXK (Zhejiang) 2021-0012). Every animal was kept in a setting with a 12-h light/dark cycle, a controlled temperature of 25 ± 2 °C, and a humidity of 55 ± 5%. Ad libitum food and drink were provided. Before testing, the rats were allowed to acclimate to the new surroundings for at least one week. The animal welfare committee of Zhejiang Chinese Medical University granted consent for animal research, and all experimental procedures complied with this approval (IACUC-20240401-08).

### Animal model of CCD

Rats in the CCD group received unilateral CCD injury. Pentobarbital (50 mg/kg i.p.) was used to anesthetize rats before exposing their transverse processes and intervertebral foramina in L4 and L5. To produce stable compression of the DRG, an L-shaped stainless steel rod measuring 0.63 mm in diameter and 4 mm in length was placed into the left L4 and L5 foramina. The incisions were subsequently closed in stages, and the rats were administered penicillin. The rats in the sham group had the identical surgical technique but did not have the L-rods inserted. Rats exhibiting autophagic abnormalities, sensory impairments, or disabilities were excluded from the study.

### PWT measurement

The PWT was obtained on base (before CCD) and on 1 day, 3 days, 5 days, and 7 days after CCD. Before the baseline test, rats were acclimated to the test environment daily for three days. Before testing, each rat was acclimated for 30 min in clear plexiglass chambers set up on an elevated mesh floor. The mid-plantar surface of the rat's hind paw was attached with von Frey filaments to allow the researchers to quantify mechanical pain thresholds. The recorded stimulus value was computed using the up-and-down [26] approach.

### Spontaneous pain behavior

As described in our earlier paper [12], rats were acclimatized in separate standard cages for 1 h before EA. The assessment of spontaneous pain perception commenced immediately following the administration of α,β-me ATP. For ten minutes, the animals were housed in Plexiglas enclosures above an HD camera (Sony, HDRCX405). The frequency of paw flinches was measured in order to evaluate persistent spontaneous nociception (PSN). Every two minutes, the number of paw flinches was recorded. Over the course of the 10-min test, paw flinches at 2-min intervals were used to capture pain-like behavior, such as shaking, biting, and licking the injected paw. Flinching responses were measured and examined to evaluate the nociceptive response caused by α,β-meATP injection.

### Drug administration

As previously disclosed [12], α β-me ATP (Sigma-Aldrich, M6517-5MG) was freshly dissolved in sterile 0.9% physiological saline and diluted to the necessary concentration prior to each experiment. Intraplantar injection of α,β-me ATP (50 μL, 100 nmol) was performed in the left hind paw. Equal amounts of sterile saline were given to the other groups. Dynamically, baseline and postoperative days after surgery were examined for mechanical pain behaviors and α,β-meATP-induced flinching behavior.

### Western blotting

On the seventh day of behavioral testing, the animals were sacrificed. Pentobarbital (50 mg/kg, i.p.) was used to completely sedate the rats, and 150 mL of normal saline (4 °C) was perfused transcardially. The DRG's L4-5 segments were removed and stored at – 80 °C. The DRG tissues were pulverized and centrifuged in a RIPA lysis solution. The upper liquid component was collected, and the amount of protein was quantified using the BCA procedure. Electrophoresis was used to separate the DRG proteins, which were then transferred to the PVDF membrane. The membranes were sealed with a 5% nonfat milk solution for one hour at room temperature. The primary antibodies were then incubated for 16 h at 4 °C before the secondary antibodies were injected for 2 h at room temperature. The following antibodies were used in this experiment: rabbit anti-P2X3 (1:1000, APR-016, Alomone), rabbit anti-TNF-α (1:1000, #29084, SAB), rabbit anti-IL-1β (1:1000, #41059, SAB), and anti-rabbit IgG, HRP-linked (1:5000, #7074s, CST). Mouse anti-β-actin (1:5000, #3700s, CST) served as a reference control. The gel images were subjected to densitometry analysis using the ImageJ software (NIH, Bethesda, MD, USA).

### Immunofluorescence

Rats were given an intraperitoneal sodium pentobarbital dose of 50 mg/kg to completely sedate them. After that, they were perfused with saline and 4% PFA in PBS 1X (pH 7.4). Following extraction and post-fixation in 4% PFA for four hours, the bilateral L4-5 DRGs were dehydrated for 48 h in a 15% and 30% sucrose solution (until sinking at 4 °C was observed). Using a frozen microtome, tissues were sectioned at a thickness of 10 μm and arranged on glass slides. Sections were blocked with 10% normal goat serum in TBST (0.1% Tween-20) for 60 min at 37 degrees Celsius after being cleaned with TBST (pH 7.4). The primary antibodies were as follows: rabbit anti-P2X3R (1:400; Alomone Labs, Jerusalem, Israel), pig anti-TRPV1 (1:200; ACC030-GP, Alomone, Israel), rabbit anti-SP (1:1000; ab67006, Abcam, UK), and rabbit anti-CGRP (1:1000; #14959, Cell Signaling Technology). The slides were removed from the refrigerator the next day, washed to remove the primary antibodies, and incubated for one hour at 37 °C with the appropriate secondary antibodies before being counterstained with DAPI. This experiment employed the following secondary antibodies: Alexa Fluor 594 goat anti-rabbit IgG (1:400, ab150084, Abcam) and Alexa Fluor 488 goat anti-guinea pig IgG (1:200, ab150185, Abcam). Photographs were captured under identical conditions, and during the quantification process, researchers were kept unaware of the treatment groups. As previously indicated, 5–8 randomly selected images from each rat tissue group were averaged and compared.

### Electroacupuncture

The EA intervention began after the behavioral test on Day 1 and continued for one week. Two stainless steel acupuncture needles, 0.18 mm in diameter and 13 mm in length, were placed to a depth of 5 mm into the same side of the body at the Zusanli (ST36) and Kunlun (BL60) acupoints. The Zusanli acupoint is 5 mm to the side of the front bump of the shinbone, while the Kunlun acupoint is at the ankle joint, between the tip of the outer ankle bone and the Achilles tendon. The current intensity varied from 0.5 to 1 mA. Specifically, the current intensity was set at 0.5 mA for the initial 15 min to allow for acclimatization, and then increased to 1 mA for the remaining 15 min of the treatment session. To compare the analgesic effects, EA was conducted at three distinct frequencies: 2 Hz, 100 Hz, and 2/100 Hz.

### Statistical analysis

GraphPad Prism 8 was used for statistical analysis, with experimental results provided as mean ± standard error (SEM). Data between two groups were compared using the Student’s t-test. Tukey’s posthoc test was used in conjunction with a one-way or two-way ANOVA to compare results between three or more groups. At a significance level of *P* < 0.05, the statistical significance was considered acceptable.

## Results

### Establishment of the rat model of CCD

**Fig. 1:**
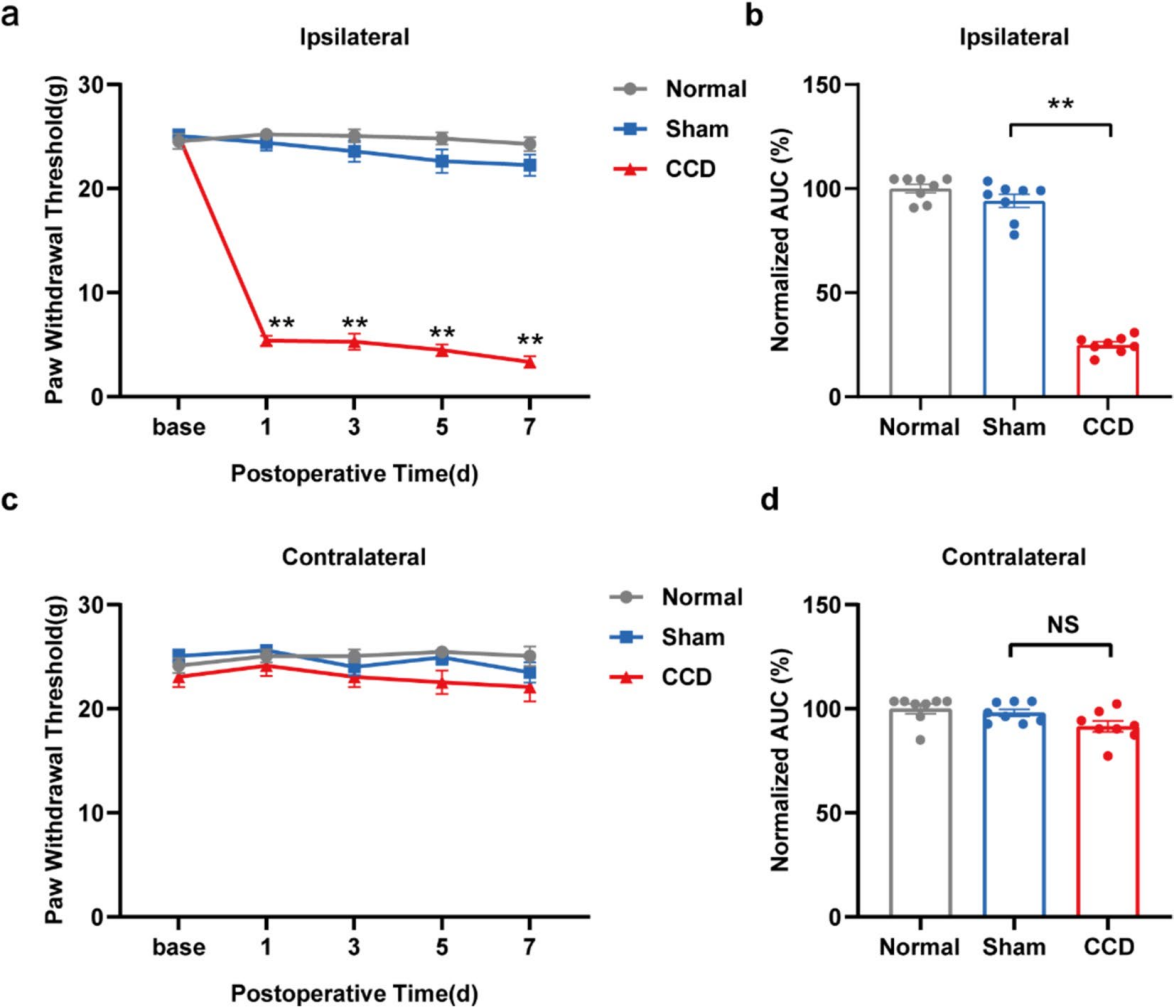
The rat CCD model exhibits unilateral, persistent, mechanically abnormal pain. **a** PWT in the ipsilateral hind paw. **b** The normalized area under the curve (AUC) is summarized in (**a**). **c** PWT in the contralateral hind paw. **d** The normalized AUC is summarized in (**c**). n = 8 rats/group. ^**^P < 0.01 compared with the Sham group. One-way or two-way ANOVA was used for statistical analysis, and Tukey's post hoc test was then run

We first established a rat CCD model based on a previously described method to mimic human radicular pain [27–29]. The result showed that the ipsilateral PWT of CCD model rats significantly decreased compared to the sham group. The ipsilateral hind limb of the CCD model rats developed significant signs of mechanical allodynia, as evidenced by a significant decrease in the ipsilateral hind PWT (Fig. 1a, b). This mechanical allodynia emerged one day after the model was built and was most pronounced on day 7. Area under the curve (AUC) analysis showed the PWT response of CCD model rats. However, there was no significant difference in mechanical pain thresholds on the contralateral side (Fig. 1c, d). These findings were similar to prior research [30], indicating the successful creation of the CCD rat model.

### The expression of P2X3 is increased in the ipsilateral DRG of CCD model rats

**Fig. 2:**
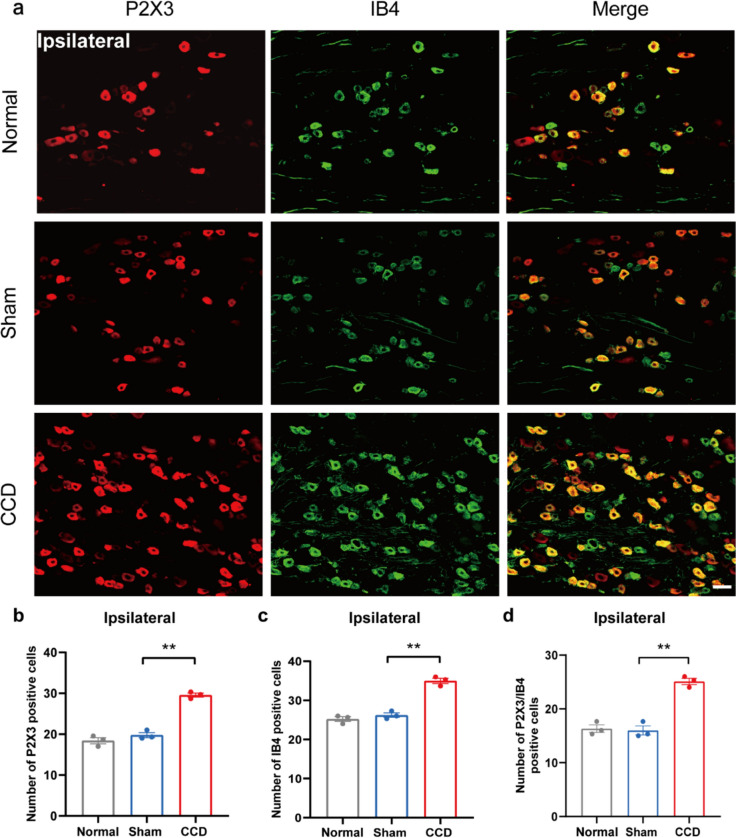
CCD increases P2X3 and IB4 expression in the ipsilateral DRG. **a** Representative immunofluorescence pictures showing co-localization of P2X3 with IB4 in the ipsilateral DRG of rats in the CCD group. **b** P2X3-positive cell count in each group's DRG. **c** IB4-positive cell count in each group's DRG. **d** P2X3/IB4 positive cell count in each group's DRG. Scale bar indicates 50 μm, n = 3 rats/group. ^**^P < 0.01 compared with the Sham group. Statistical analyses were performed by one-way ANOVA with a Tukey post hoc test

**Fig. 3:**
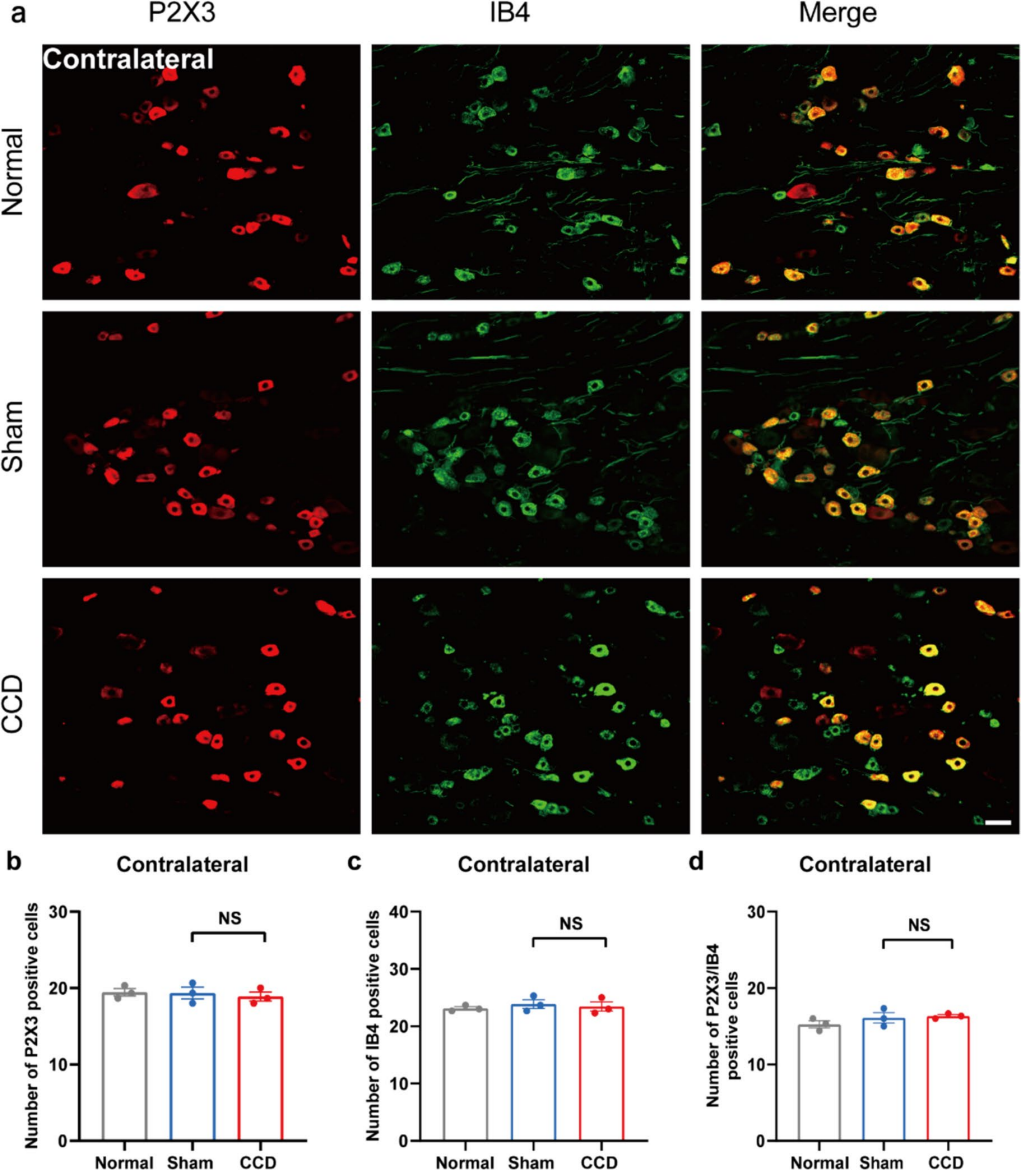
CCD did not increase P2X3 and IB4 expression in the contralateral DRG. **a** Representative immunofluorescence pictures showing P2X3 co-localization with IB4 in the contralateral DRG of rats in the CCD-7d group. **b** P2X3-positive cell count in each group's DRG. **c** IB4-positive cell count in each group's DRG. **d** P2X3/IB4 positive cell count in each group's DRG. Scale bar indicates 50 μm, n = 3 rats/group. One-way ANOVA was used in the statistical analysis, along with a Tukey post hoc test

**Fig. 4:**
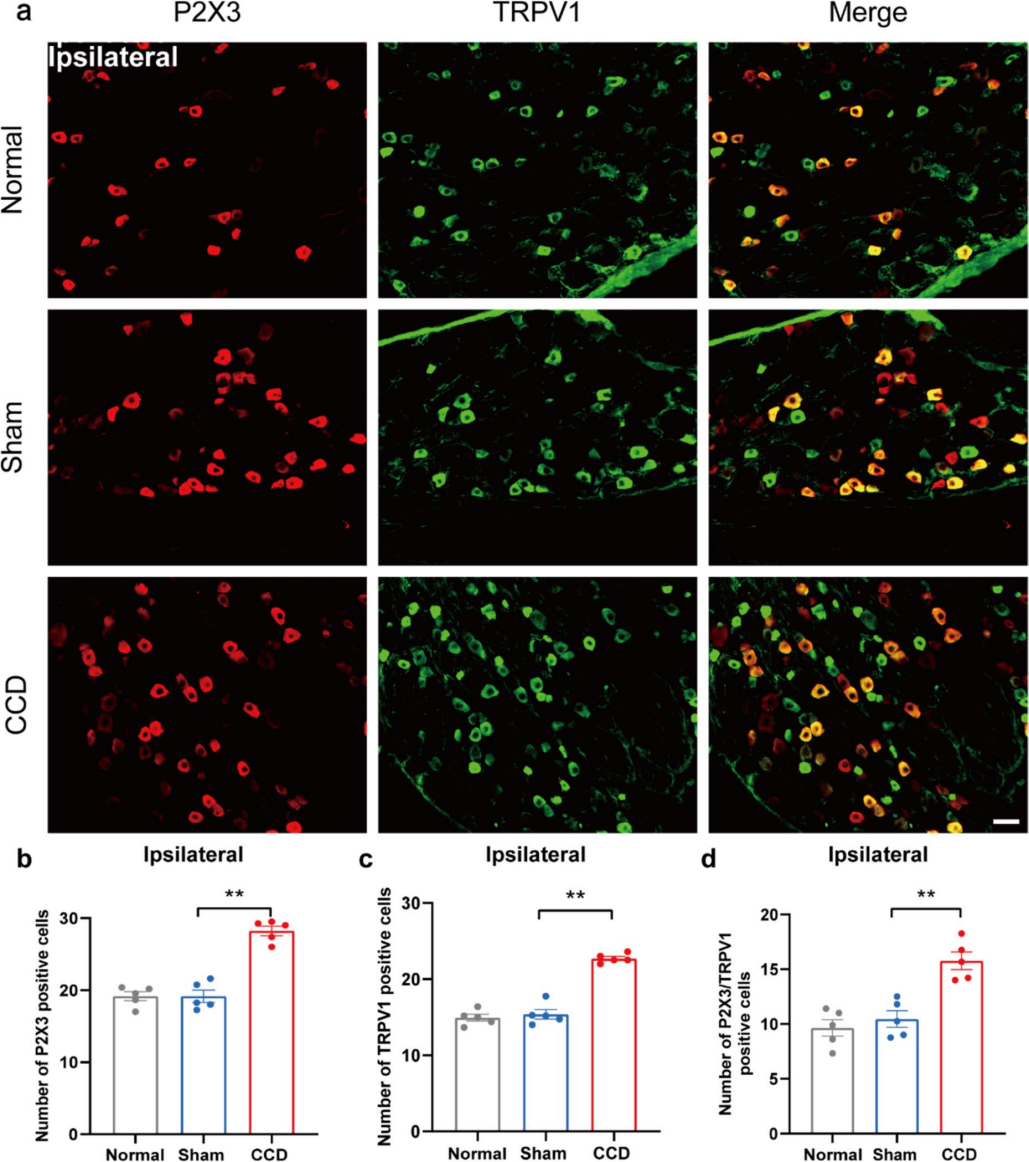
CCD increases P2X3 and TRPV1 expression in the ipsilateral DRG. **a** Representative immunofluorescence pictures showing co-localization of P2X3 with TRPV1 in the ipsilateral DRG of rats in the CCD group. **b** P2X3-positive cell count in each group's DRG. **c** TRPV1-positive cell count in each group's DRG. **d** P2X3/TRPV1 positive cell count in each group's DRG. Scale bar indicates 50 μm, n = 5 rats/group. ^**^P < 0.01 compared with the Sham group. One-way ANOVA was used in the statistical analysis, along with a Tukey post hoc test

**Fig. 5:**
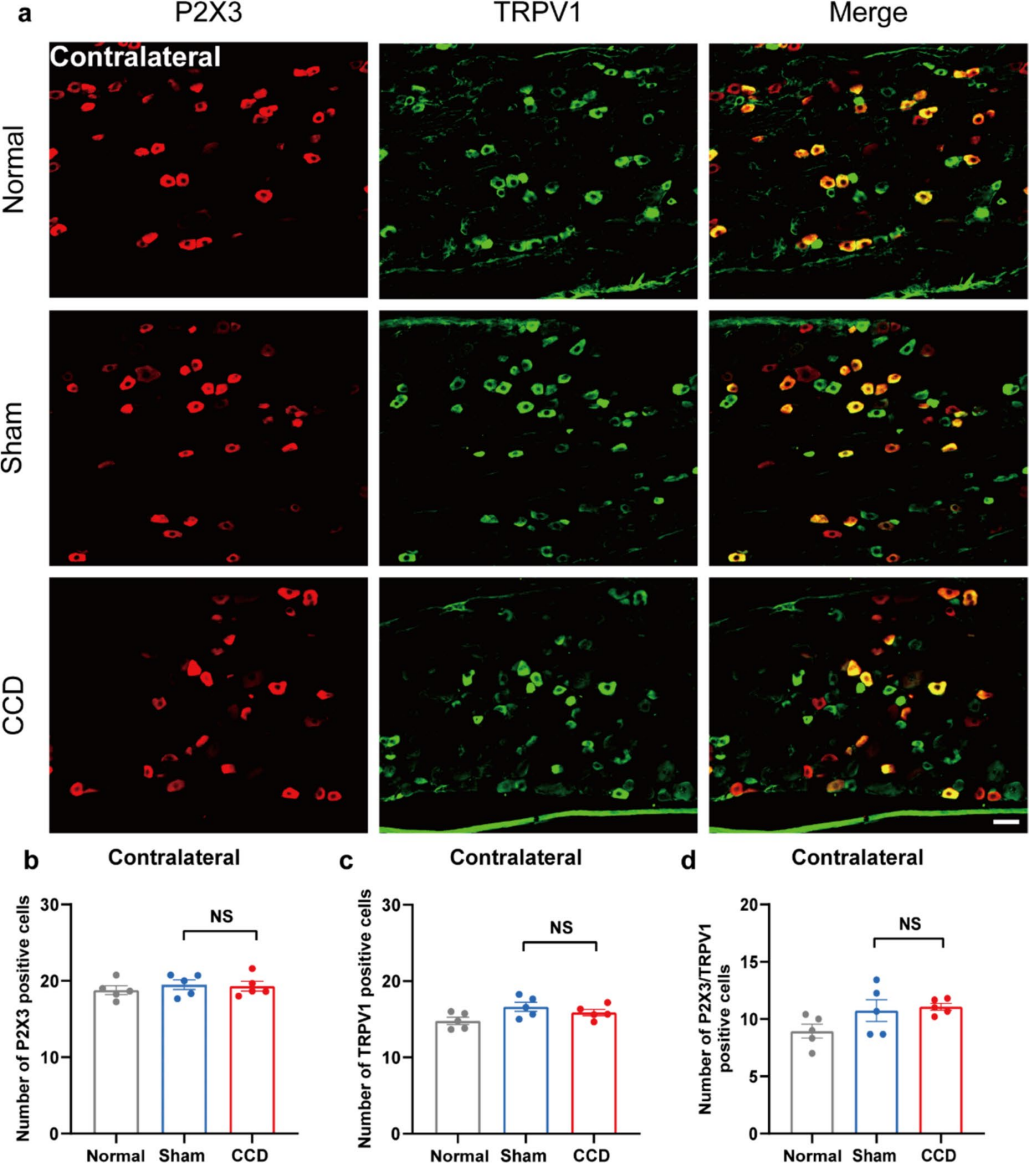
CCD did not increase P2X3 and TRPV1 expression in the contralateral DRG. **a** Representative immunofluorescence pictures showing P2X3 co-localized with TRPV1 in the contralateral DRG of rats in the CCD group. **b** P2X3-positive cell count in each group's DRG. **c** TRPV1-positive cell count in each group's DRG. **d** P2X3/TRPV1 positive cell count in each group's DRG. Scale bar indicates 50 μm, n = 5 rats/group. One-way ANOVA was used in the statistical analysis, along with a Tukey post hoc test

We examined P2X3 co-localization with IB4 and TRPV1 in DRG using double-label immunofluorescence 7 days after CCD modeling. The immunofluorescence for P2X3 with IB4 and TRPV1 in CCD rats' DRGs were comparable to those in sham rats' DRGs (Figs. 2, 3, 4, 5). The CCD group had a significantly higher number of P2X3-positive cells in the ipsilateral DRG compared to the Sham group, as well as significant co-localization with IB4 and TRPV1 (Figs. 2 and 4), suggesting sensitization of sensory neurons after chronic compression. However, no significant difference was found in the contralateral DRG between in the CCD group and Sham group (Figs. 3 and 5). In contrast, no significant difference was found in the number of P2X3, IB4, and TRPV1-positive cells in the ipsilateral and contralateral DRGs between in the sham group and Normal group.

### EA alleviates ipsilateral mechanical allodynia in CCD model rats

**Fig. 6:**
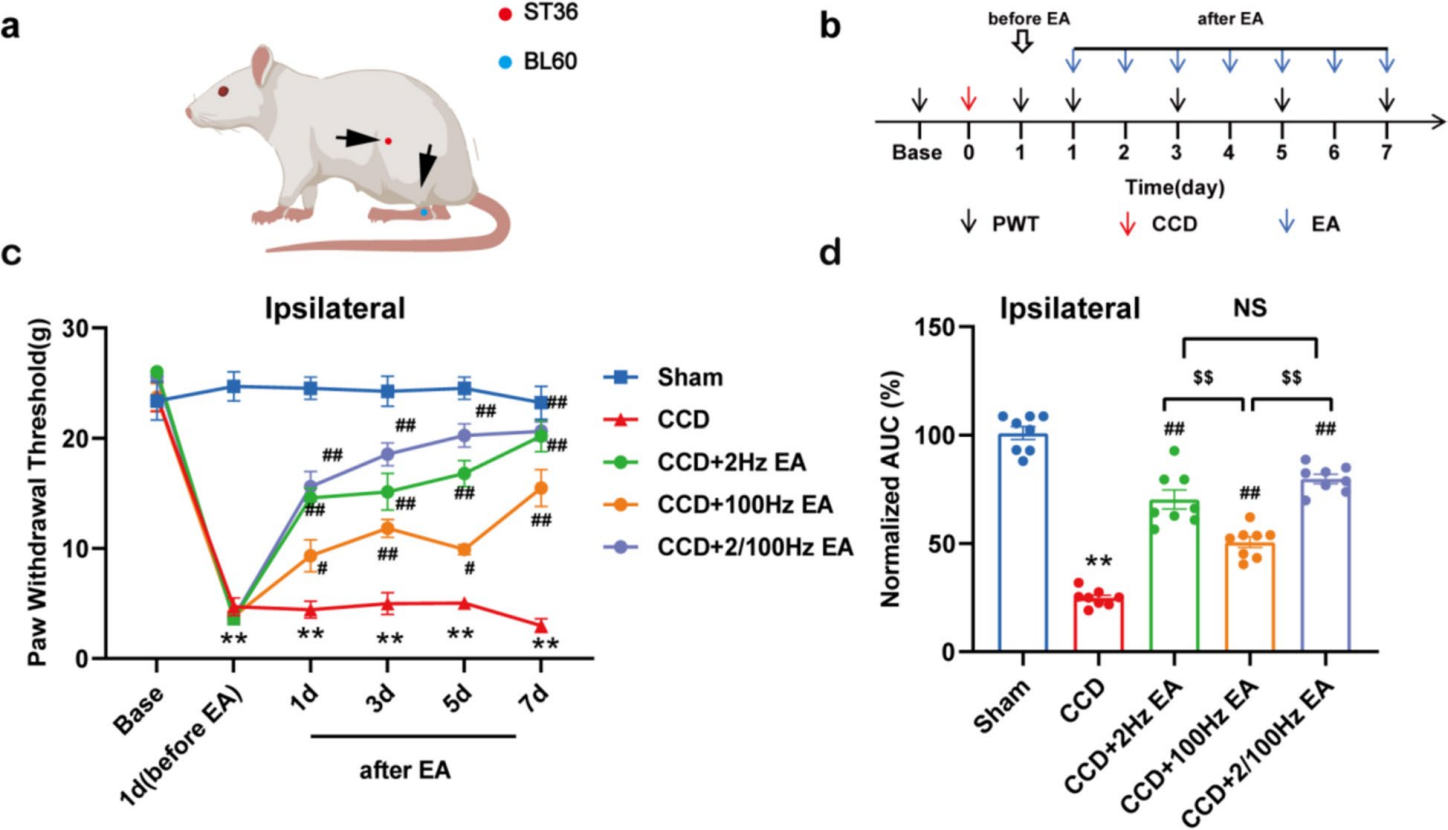
To evaluate the analgesic potential of various EA frequencies in CCD model rats. **a** A schematic representation of the ST36 and BL60 acupoints in rats. **b** An experimental protocol for EA treatment. **c** Ipsilateral 2 Hz, 100 Hz, or 2/100 Hz EA affects ipsilateral hind paw PWT in CCD rats. **d** Normalized AUC summaries as shown in (**c**). n = 8 rats/group. **P < 0.01 vs. the Sham group. ^#^P < 0.05 vs. the CCD group. ^##^P < 0.01 vs. the CCD group. ^$$^P < 0.01 vs. the CCD + 100 Hz EA group. Tukey's post hoc test was used in conjunction with one-way ANOVA for statistical analysis

Next, we investigated the effect of EA on ipsilateral mechanical allodynia in CCD model rats. Different EA frequencies may have varied therapeutic effects. We first screened for the best EA frequency to alleviate mechanically allodynia in CCD model rats. As shown in Fig. 6a, at the ST36 and BL60 locations, we first evaluated EA frequencies of 2, 100, or 2/100 Hz on the ipsilateral side of the CCD rat hindlimb. After the CCD model was established, EA was applied to it at different frequencies for 30 min per day for 7 consecutive days (Fig. 6b). The result showed that all three frequencies of EA produced different degrees of therapeutic effects on the CCD model rats. In contrast, 2 Hz and 2/100 Hz EA produced superior analgesic effects compared to 100 Hz EA, which lasted until the last EA treatment (Fig. 6c). 2/100 Hz EA has better sustained results than 2 Hz. AUC analysis further showed that 2 Hz and 2/100 Hz EA had an overall better analgesic effect compared with CCD (Fig. 6d). To maximize therapeutic effects, we selected 2/100 Hz EA as the optimal treatment option in subsequent investigations.

### EA reduced the overexpression of P2X3 in the ipsilateral DRG of CCD model rats

**Fig. 7:**
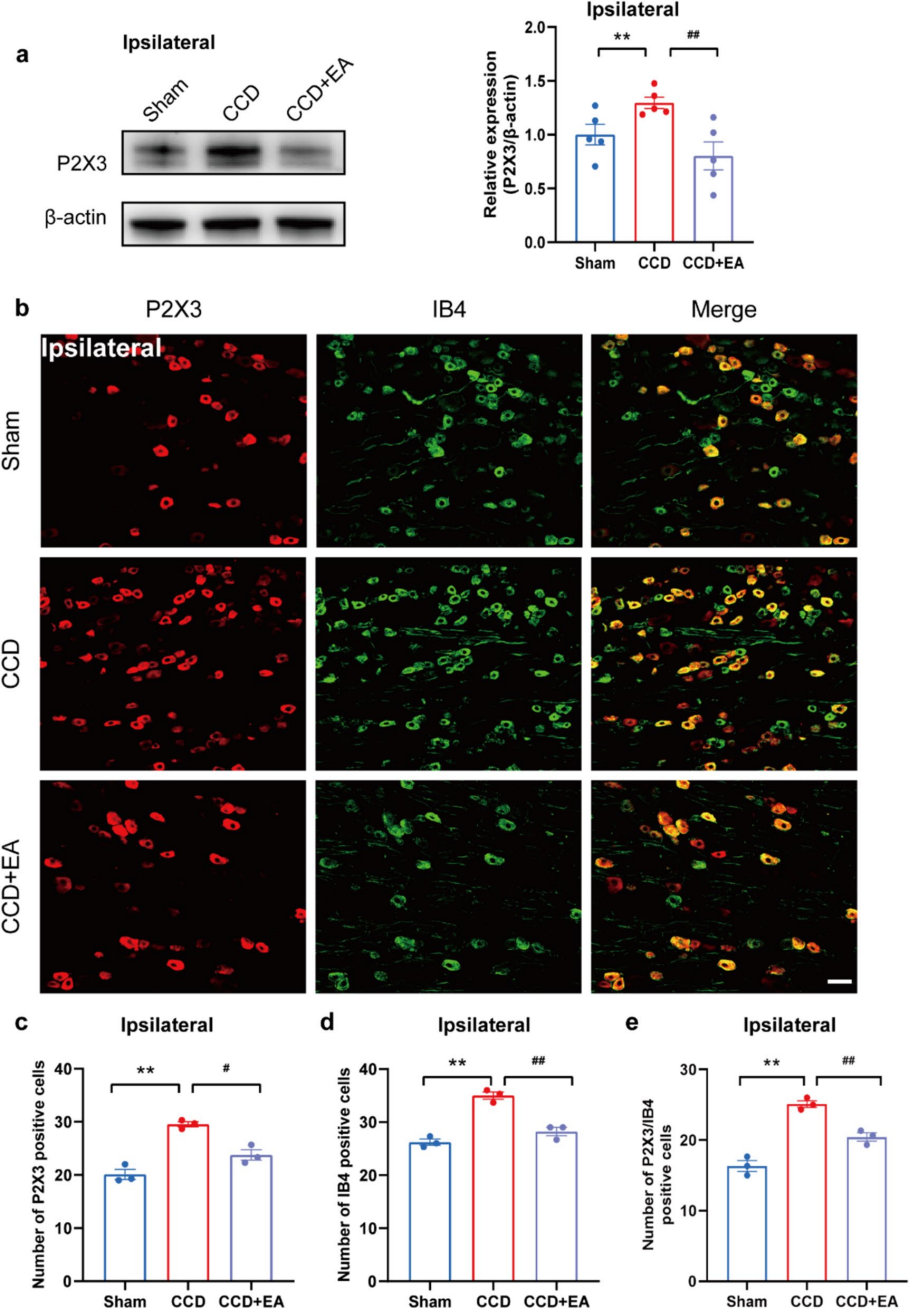
EA inhibits the expression of P2X3 and IB4 in the DRG of CCD. **a** Western blot showing protein expression of P2X3. **b** Representative immunofluorescence pictures demonstrating P2X3 and IB4 co-localization in the ipsilateral DRG. **c** P2X3-positive cell count in each group's DRG. **d** IB4-positive cell count in each group's DRG. **e** P2X3/IB4 positive cell count in each group's DRG. Scale bar indicates 50 μm, n = 3/group. ^**^P < 0.01 vs. the Sham group. ^#^P < 0.05 vs. the CCD group. ^##^P < 0.01 vs. the CCD group. To do statistical analysis, Tukey's post hoc tests and one-way ANOVA were used

**Fig. 8:**
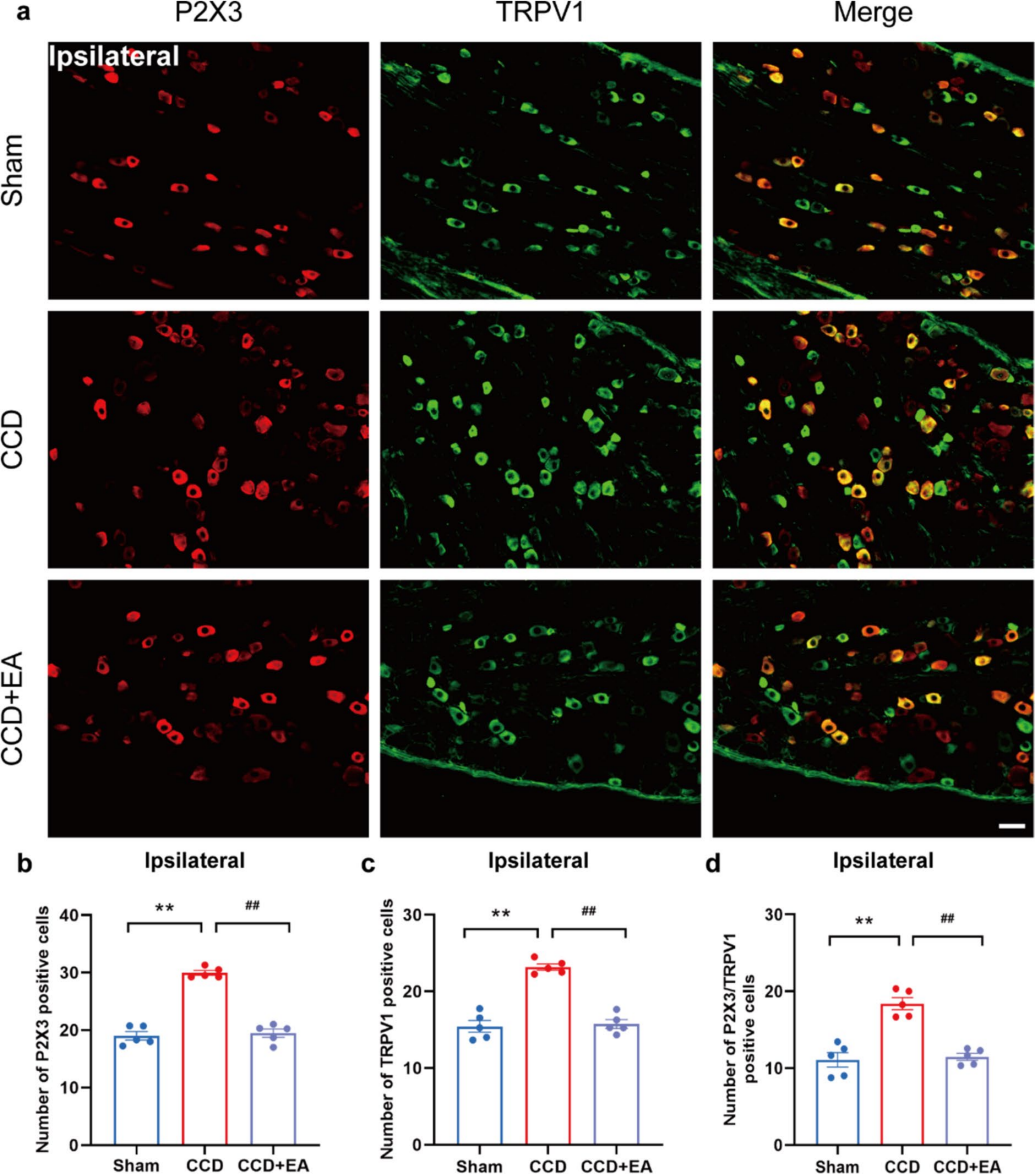
EA inhibits the expression of P2X3 and TRPV1 in the ipsilateral DRG of CCD. **a** Representative immunofluorescence pictures showing the co-localization of P2X3 with TRPV1. **b** P2X3-positive cell count in each group's DRG. **c** TRPV1-positive cell count in each group's DRG. **d** P2X3/TRPV1 positive cell count in each group's DRG. Scale bar indicates 50 μm, n = 5/group. ^**^P < 0.01 vs. the Sham group. ^##^P < 0.01 vs. the CCD group. Tukey's post hoc test and one-way ANOVA were used in the statistical analysis

Western blot analysis revealed that EA treatment significantly decreased the overexpression of P2X3 protein in the ipsilateral DRG of CCD model rats compared with the CCD group (Fig. 7a). Immunofluorescence further analysis revealed that EA therapy significantly reduced the overexpression of P2X3, IB4, and TRPV1 in the ipsilateral DRG of CCD model rats compared to CCD (Figs. 7, 8). Specifically, the number of P2X3-positive cells after EA treatment compared to the untreated CCD group led to a significant reduction in P2X3 immunoreactivity (Figs. 7c and 8b). Accompanied by a decreased number of IB4 and TRPV1-positive cells and co-localization of P2X3 with both IB4 and TRPV1, indicative of attenuated sensory neuron sensitization following EA therapy. These findings suggest that EA may exert analgesic effects on CCD-induced neuropathic pain by modulating P2X3 receptor expression and activity, as well as through interactions with IB4 and TRPV1.

### EA reduced the overexpression of CGRP, SP, and inflammatory cytokines in the ipsilateral DRG of CCD model rats

**Fig. 9:**
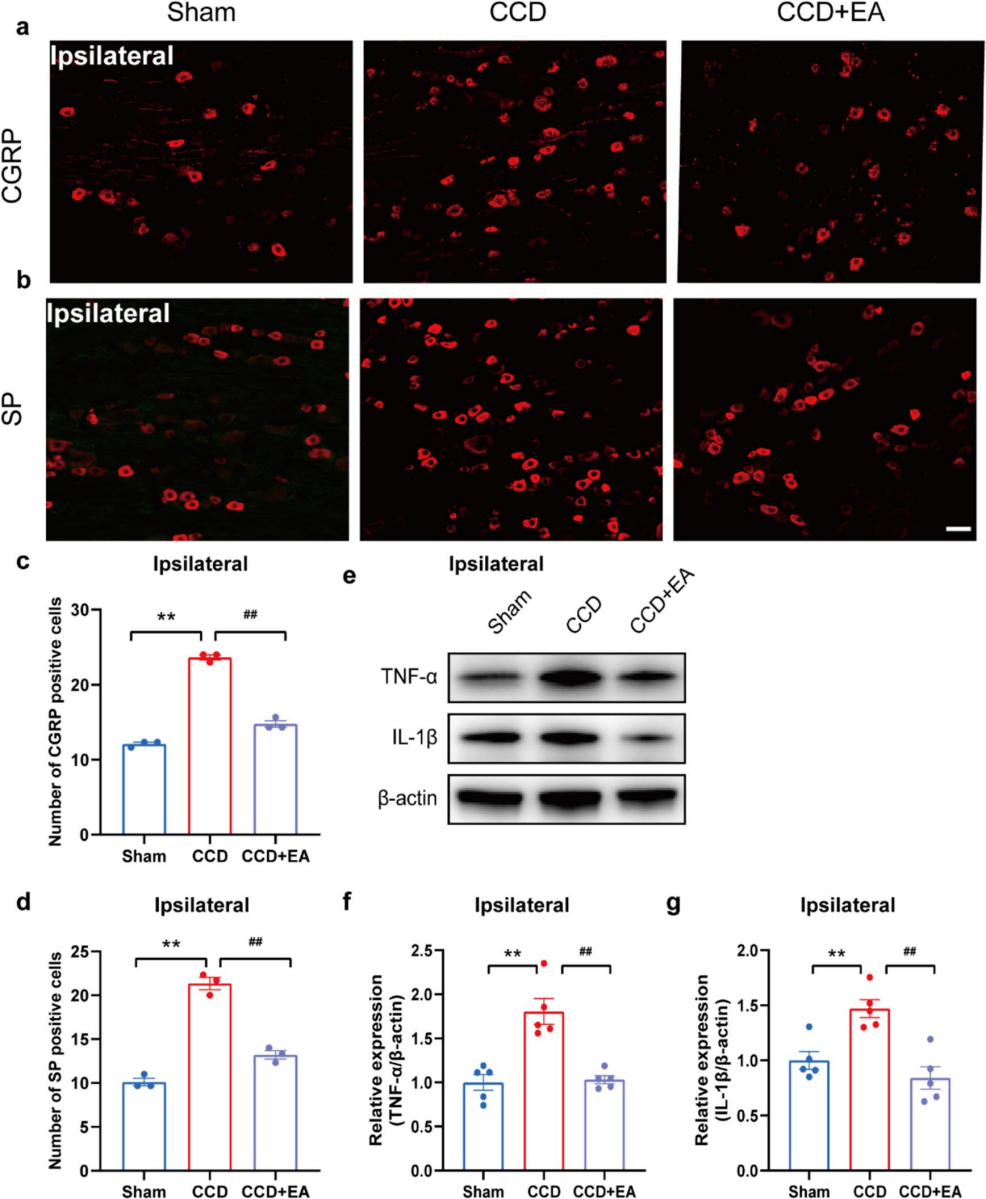
EA inhibits the expression of CGRP, SP, and inflammatory cytokines in the ipsilateral DRG of CCD. **a** Representative immunofluorescence images showing positive cellular expression of CGRP in the ipsilateral DRG of rats in the CCD group. Scale bar indicates 50 μm, n = 3 rats/group. **b** Representative immunofluorescence images showing positive cell expression of SP in the ipsilateral DRG of rats in the CCD group. Scale bar indicates 50 μm, n = 3 rats/group. **c** Number of CGRP-positive cells in the DRG of each group. **d** The number of SP-positive cells in the ipsilateral DRG of each group. **e**–**g** TNF-α and IL-1β protein expression in ipsilateral DRG is demonstrated using a western blot. ^**^P < 0.01 vs. the Sham group. ^##^P < 0.01 vs. the CCD group. Tukey's post hoc test and one-way ANOVA were used for statistical analysis

Next, we investigated the possibility that the modification of CGRP and SP signaling in DRG was connected to the analgesic effect that EA generated in CCD model rats. Immunostaining revealed that EA intervention resulted in a marked decrease in SP and CGRP expression in the ipsilateral DRG of CCD rats (Figs. 9a–d), reflecting a suppression of neuropeptide-mediated nociceptive signaling pathways associated with neuropathic pain. Additionally, EA also reduced the expression of pro-inflammatory cytokines such TNF-α and Il-1β. WB indicated that TNF-α and Il-1β levels were significantly up-regulated in ipsilateral DRG of CCD group vs. Sham group (Figs. 9e–g). EA intervention significantly reduced the expression of TNF-α and Il-1β in ipsilateral DRG (Figs. 9e–g). These findings suggest that EA may exert its analgesic effects in CCD-induced neuropathic pain, by inhibiting SP and CGRP expression in sensory neurons and regulating inflammatory cytokines.

### P2X3 inhibition contributed to the analgesic effects of EA on CCD-induced neuropathic pain

**Fig. 10:**
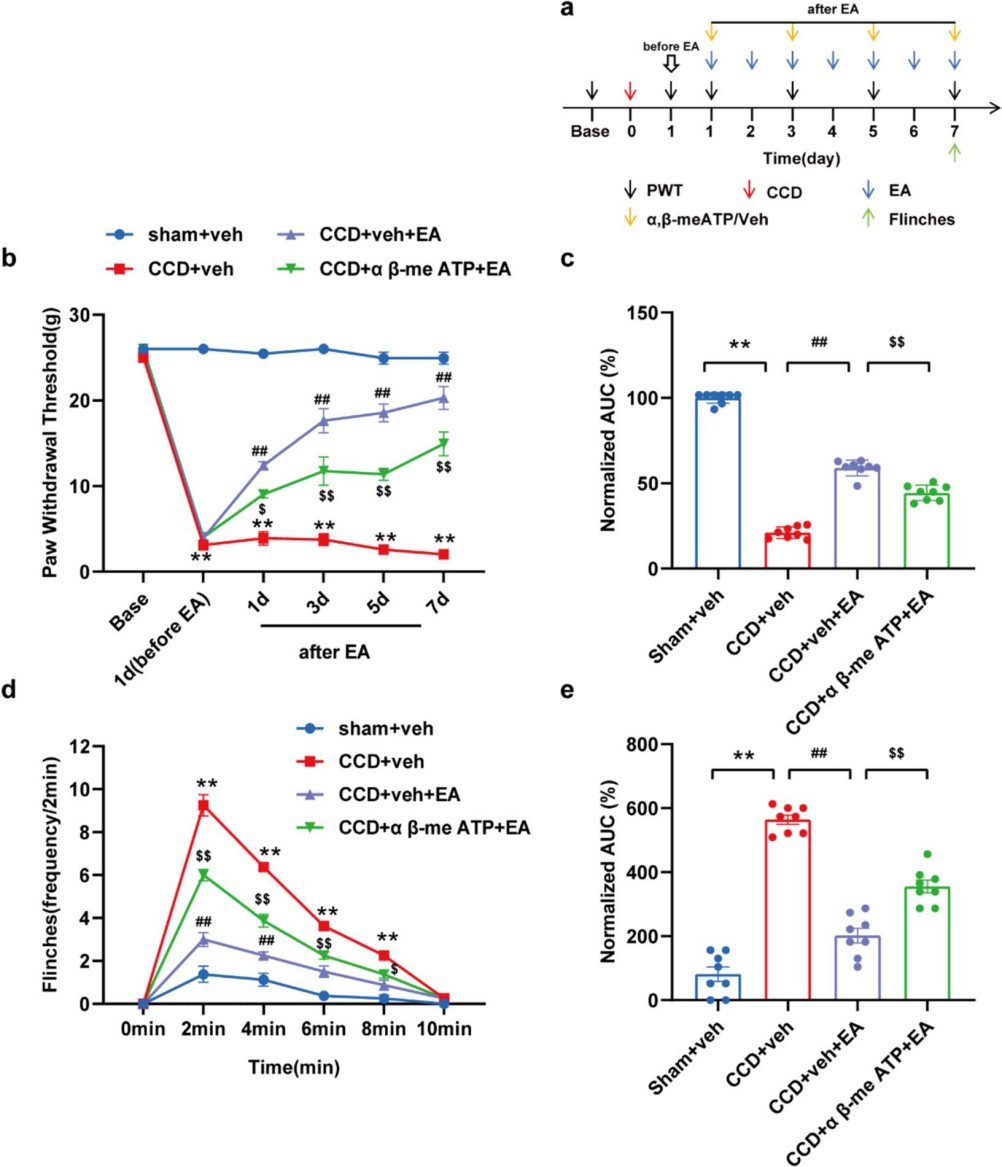
α,β-me ATP (P2X3 agonist) attenuated the analgesic effect of 2/100 Hz EA in CCD rats. **a** Experimental timeline showing time points of PWTs, model construction, EA intervention, P2X3 agonist α,β-meATP intervention, and flinch test. **b** On days 1, 3, 5, and 7 following inoculation, PWTs of CCD + α,β-meATP + EA rats were lower than those of CCD + veh + EA rats. **c** AUC, D1–7 analysis (n = 8/group). **d** 1–4 min following α,β-me ATP injection into the plant on day 7, the frequency of withdrawal was higher in CCD + α,β-me ATP + EA rats than in CCD + veh + EA rats. **e** AUC analysis (0–10 min, n = 8/group). Data are expressed as mean ± SEM compared with the CCD + veh + EA group at the same time point. ^**^P < 0.01.vs. the Sham + veh group. ^##^P < 0.01. vs. the CCD + veh group. ^$^P < 0.05, ^$$^P < 0.01.vs. the CCD + veh + EA group. One-way or two-way ANOVA was used for statistical analysis, and Tukey's post hoc test was then run

**Fig. 11:**
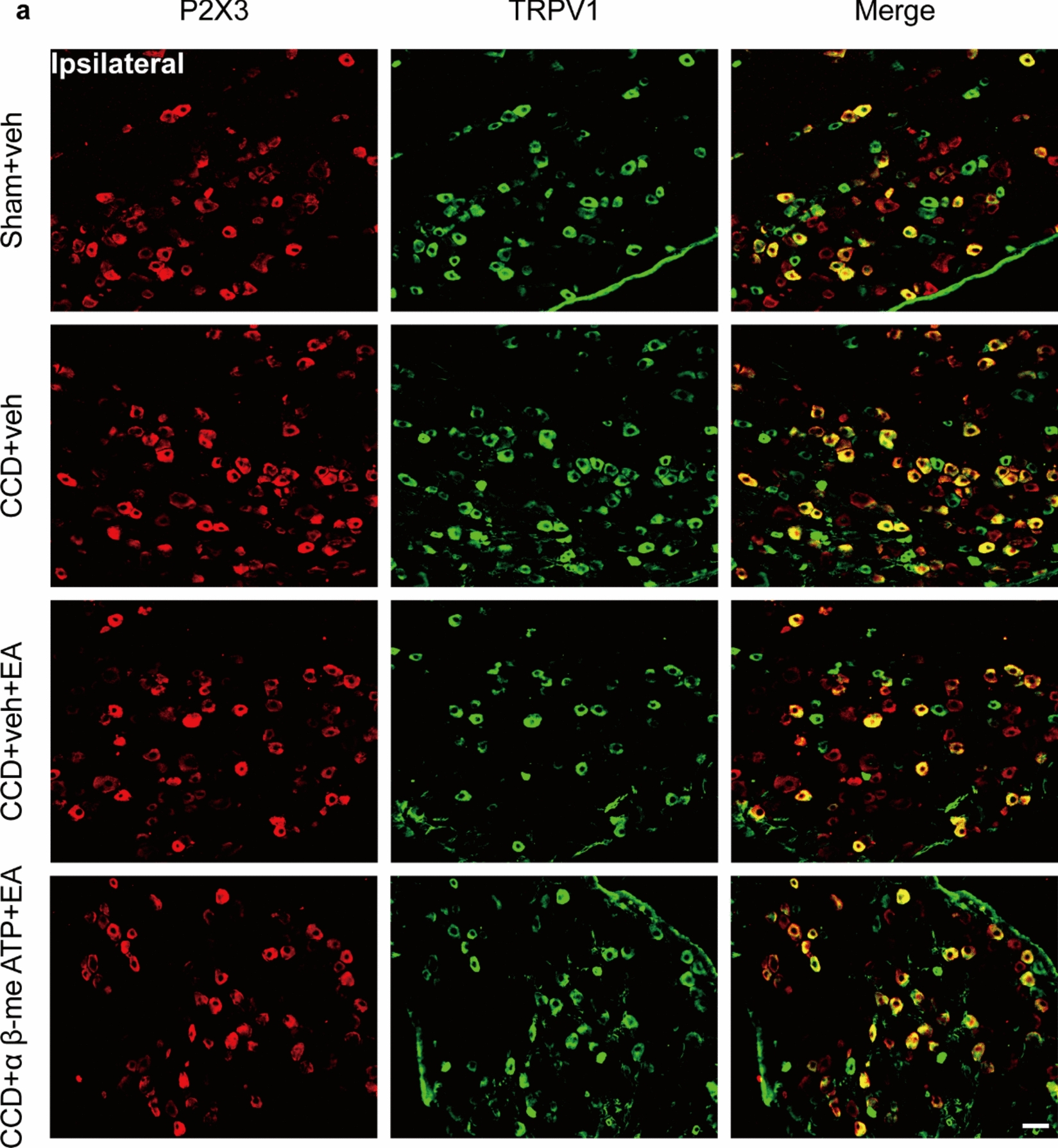

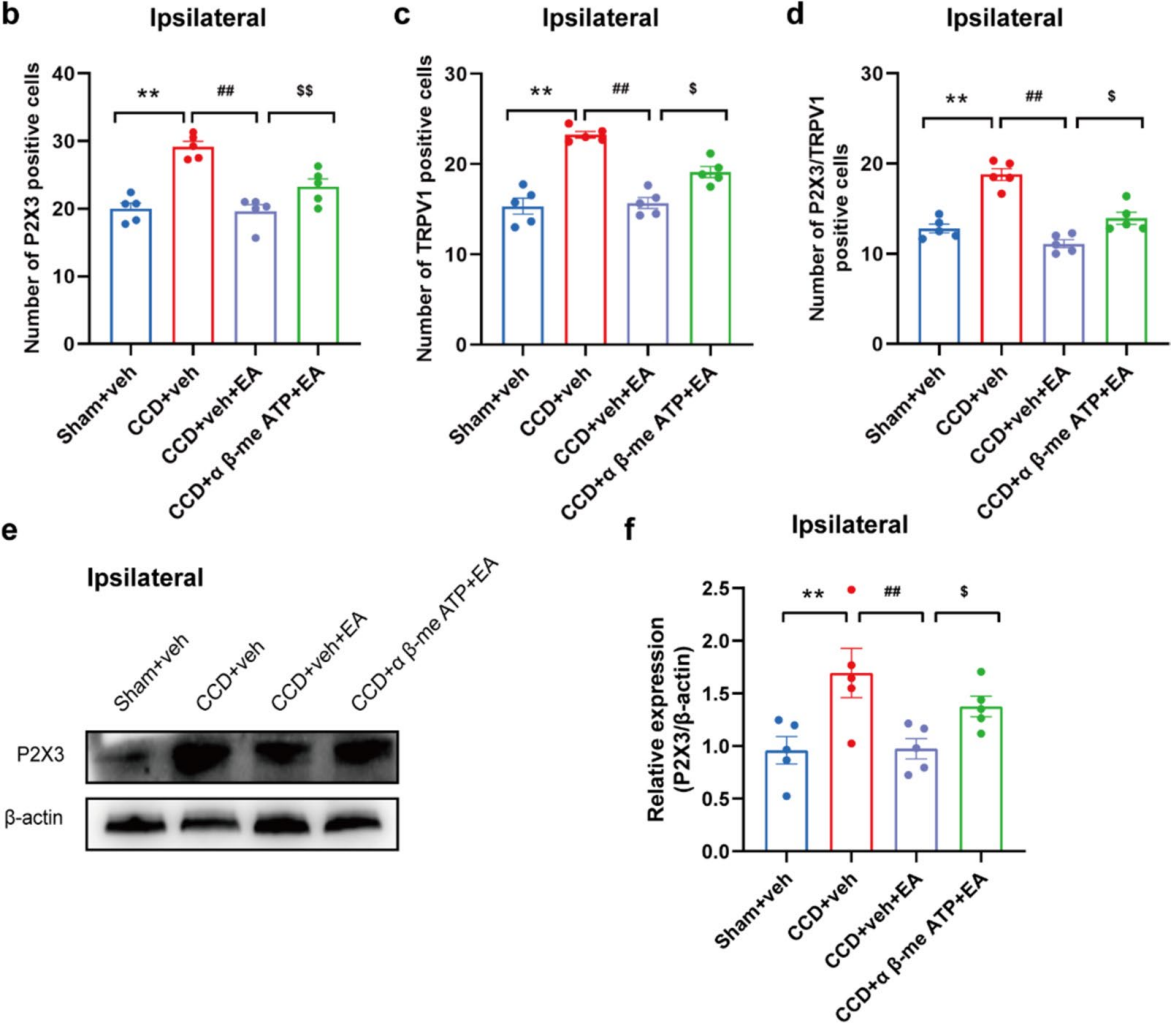
α,β-me ATP attenuated the analgesic effect of 2/100 Hz EA in CCD rats. **a** Representative immunofluorescence pictures showing the co-localization of P2X3 with TRPV1. **b** P2X3-positive cell count in each group’s DRG. **c** TRPV1-positive cell count in each group’s DRG. **d** P2X3/TRPV1 positive cell count in each group's DRG. Scale bar indicates 50 μm, n = 5/group. **e**, **f** P2X3 protein expression in DRG is demonstrated using a western blot. n = 5/group. **P < 0.01 vs. the Sham + veh group.. ^##^P < 0.01 vs. the CCD + veh group. ^$^P < 0.05, ^$$^P < 0.01 vs. the CCD + veh + EA group. Tukey’s post hoc test and one-way ANOVA were used in the statistical analysis

Next, we assessed the effect of EA on P2X3-mediated activity. Previous results showed that CCD model rats developed mechanical allodynia on the first day after modeling. EA treatment was started on day 1 and continued for seven days in a row. α,β-meATP, a specific P2X3 agonist, was injected before EA stimulation every other day. Then track the variations in PWT among the rats in each group. α,β-me ATP was administered into the rat's ipsilateral foot plantar surface instantly. After EA treatment on day 7 to activate the P2X3, which triggered persistent spontaneous nociceptive sensation (PSN) (Fig. 10a). Figure 10b shows that after injecting α,β-meATP, EA had a lesser analgesic effect on CCD compared to rats given the vehicle. Furthermore, AUC analysis revealed that P2X3R activation decreased the anti-allodynic effect of EA stimulation in CCD rats (Fig. 10c). After injection of α,β-me ATP, the number of paw retractions was significantly increased in rats in the EA group. The CCD + α,β-meATP + EA group elicited more retractions than the CCD + Veh + EA group, particularly 4 min after plantar injection (Fig. 10d). AUC analysis revealed that the P2X3R agonist effectively reversed the analgesic effect of EA on α,β-meATP-induced spontaneous pain (Fig. 10e). Immunofluorescence staining revealed that P2X3 receptor expression was increased in ipsilateral DRG of the CCD + α,β-meATP + EA group rats compared to the CCD + EA + veh group (Figs. 11a–d). WB analysis corroborated these findings, showing upregulation of P2X3 protein levels in the ipsilateral DRG of the CCD + α,β-meATP + EA group compared to the CCD + veh + EA group (Fig. 11e).

## Discussion

In this study, we established a rat CCD model simulating clinical sciatica and low back pain. Our findings demonstrate that EA exerts significant analgesic effects, as evidenced by the attenuation of mechanical allodynia in CCD rats. Moreover, our results reveal that different frequencies of EA stimulation (2 Hz, 100 Hz, and 2/100 Hz) exert varying degrees of analgesic effects, with the 2/100 Hz EA regimen demonstrating superior efficacy compared to 100 Hz stimulation. We found that EA treatment eliminated mechanical allodynia and suppressed P2X3R overexpression. This differential response to EA frequency underscores the importance of optimizing stimulation parameters to maximize therapeutic outcomes [10]. More importantly, the P2X3 agonist α,β-me ATP attenuated the analgesic effect of 2/100 Hz EA in CCD rats. These results imply that increased P2X3R expression may promote CCD and that EA may function as an analgesic by preventing P2X3R expression in the DRGs. Our study shows that 2/100 Hz EA reduces P2X3R overexpression and functional activity in CCD rat DRGs. By considering all these findings together, we identified a potential analgesic target and demonstrated that 2/100 Hz EA could alleviate CCD by inhibiting P2X3 expression, thus providing a new idea for clinical treatment of CCD.

The most prevalent type of neuropathic pain is neurogenic neuralgia, which arises when unpleasant factors irritate the basal bones of the spine or the DRG, such as lumbar disc herniation, lumbar spinal stenosis, spinal cord tumor compression, and certain inflammatory compounds. Sciatica and low back pain are frequently brought on by chronic compression of the DRG or its proximal nerve roots after a spinal injury, disc herniation, or foraminal stenosis [31]. Sciatica and low back pain are two of the most common types of neuropathic pain in clinical practice [32]. Rodent models of CCD are important tools for studying neuropathic pain mechanisms and evaluating potential therapeutic interventions for clinical conditions such as sciatica and low back pain [33]. Neuropathic pain caused by these two pain conditions is usually ineffective against conventional pharmacological treatments, and therefore novel therapeutic approaches are urgently needed [34]. The CCD model employed in our work may advance our understanding of the neurological underpinnings of chronic compression-induced low back pain and sciatica in DRG, helping create targeted therapies [26].

EA has been globally recognized as an effective treatment for a wide range of clinical pains, including musculoskeletal pain [35], cancer pain [36], dental pain [37], visceral pain [38] and labor pain [39]. According to TCM theory, the acupoint Zusanli (ST36), located on the Stomach meridian, is traditionally used to strengthen the body's Qi and Blood, and alleviate pain in the limbs. Kunlun (BL60), situated on the Bladder meridian, is noted for its effectiveness in treating lower limb pain, paralysis, and soft tissue disorders. Both are commonly used points for analgesia in clinical settings. Previous studies has demonstrated that EA at ST36 and BL60 significantly reduces inflammatory and neuropathic pain [15, 25, 40]. However, numerous investigations have revealed that different frequencies of EA stimulation elicit varied analgesic effects and processes [21, 23]. Our previous investigations showed that EA had a substantial analgesic impact on DNP in rats [41, 42] and is affected by different treatment frequencies [22]. Some research [43] has shown that different stimulation periods (20, 30, 45 min) and frequencies (2 Hz, 100 Hz, 2/100 Hz) of EA had analgesic effects on the PWT and PWL following CFA injection. However, the frequency of EA was the main factor that correlated with its degree of hypoalgesic efficacy. The analgesic effect is more pronounced at 100 Hz compared to 2 Hz for inflammatory pain in rats. Similarly, other studies have concluded that both 2 Hz and 100 Hz EA can alleviate neurogenic pain, with 2 Hz EA demonstrating superior analgesic efficacy compared to 100 Hz [23]. Our behavioral study showed that EA treatment attenuated the mechanically abnormal pain induced by CCD. We investigated the best EA frequency for treating mechanical hyperalgesia in CCD model rats. The level of analgesia in CCD model rats varied at three different EA frequencies during the treatment period. 2/100 Hz EA and 2 Hz EA had a stronger analgesic effect than 100 Hz EA. Therefore, we chose EA at a frequency of 2/100 Hz to maximize the analgesic effect [44].

P2X3 receptors play an essential role in nociceptive signaling and neural sensitization, with the increase seen in response to nerve injury and inflammation [11]. Consistent with previous reports, our findings demonstrate an increase in P2X3 expression in the ipsilateral DRG of CCD rats, indicative of sensory neuron sensitization following peripheral nerve injury [2]. We suggest that PWT and immunohistochemical markers were predominantly affected ipsilaterally in the CCD model, where chronic compression was applied. It is consistent with previous research indicating that neuropathic pain induced by localized injury, such as in the CCD model, primarily affects the ipsilateral side [29, 30, 45, 46]. Recent research suggests that EA's analgesic action is directly related to its modulation of ion channels in sensory neurons [47, 48]. P2X3, a possible target of inflammatory and neuropathic pain, has received a lot of interest [49]. Importantly, EA intervention significantly reduced P2X3 expression, suggesting its potential as a therapeutic strategy for modulating purinergic signaling pathways implicated in neuropathic pain [14]. In this work, we showed that α,β-meATP-induced mechanical hyperalgesia, and nocifensive behavior were facilitated by CCD injuries. P2X3 is thought to be the main mediator of mechanical hyperalgesia and nocifensive flinch responses in small and medium nociceptors that are triggered by ATP or its analogue, α,β-meATP [50, 51]. The hyperexcitability of DRG neurons is responsible for the increased pain behavior of α,β-meATP [52]. P2X3 receptors in this study are predominantly detected in tiny nociceptive DRG neurons that have undergone CCD. Consequently, increased P2X3 receptor expression and larger α,β-meATP responses in small-sized DRG neurons could account for the increased behavioral sensitivity to α,β-meATP injection in the CCD-injured state.

In addition to P2X3 receptor modulation, our study revealed enhanced expression of IB4 and TRPV1, markers of nociceptive sensory neurons, in the CCD rats' DRG. IB4-positive neurons represent a subpopulation of non-peptidergic nociceptive neurons involved in transmitting pain signals. TRPV1 acts as a molecular transmitter of unpleasant stimuli like heat and inflammatory mediators [53]. The observed increase in IB4 and TRPV1 expression further corroborates the sensory neuron sensitization characteristic of neuropathic pain conditions [4]. We observed IB4-positive labeling with granular cytoplasm and intense plasma membrane staining. IB4 binding was limited to small and medium-sized neurons. IB4 is bound by the majority of tiny p2 × 3-positive neurons. P2X3 and TRPV1 were widely co-expressed in DRG neurons with small and medium diameters, similar to our previous work, which showed a relationship between TRPV1 and P2X3 using co-immunofluorescence [46]. Notably, EA treatment attenuated the overexpression of IB4 and TRPV1 in the DRG of CCD rats, suggesting its ability to modulate the activity of nociceptive sensory neurons and mitigate pain hypersensitivity [54].

Furthermore, our study elucidated the role of neuropeptides such as SP and CGRP in neuropathic pain modulation by EA. SP and CGRP are key mediators of neurogenic inflammation and nociceptive transmission, contributing to peripheral and central sensitization in neuropathic pain states [55]. Consistent with previous reports, our findings demonstrate elevated SP and CGRP expression in the DRG of CCD rats, indicative of neuropeptide-mediated nociceptive signaling [1]. Remarkably, EA intervention resulted in the downregulation of SP and CGRP expression, suggesting its potential to attenuate neurogenic inflammation and nociceptive transmission associated with neuropathic pain [56]. We also investigated the effects of EA on the TNF-α and IL-1β expression levels in CCD rat DRG. Our WB analysis revealed that TNF-α and IL-1β protein levels were significantly elevated in the DRG of CCD model rats, indicating an inflammatory response associated with neuropathic pain. EA treatment effectively downregulated the expression of these pro-inflammatory cytokines. These findings suggest that EA exerts its analgesic effects, at least in part, by modulating inflammatory pathways in the DRG and underscore the potential of EA as a therapeutic intervention for inflammation-mediated neuropathic conditions [57, 58]. Further studies are warranted to elucidate the precise signaling pathways through which EA modulates cytokine expression and to explore its long-term efficacy and safety in clinical settings.

In summary, our study provides mechanistic insights into the analgesic effects of EA in neuropathic pain management, highlighting its ability to modulate purinergic receptor signaling, sensory neuron activity, neuropeptide expression, and inflammatory cytokine expression in the DRG. By targeting multiple components of the pain pathway, EA offers a comprehensive approach to alleviating neuropathic pain symptoms, with implications for the development of personalized and effective treatments for patients with conditions such as sciatica and lower back pain.

## Conclusions

Our research shows that ipsilateral 2 Hz EA, 100 Hz EA and 2/100 Hz EA can effectively elicit an antiallodynic effect in a rat model of CCD. 2/100 Hz showing superior efficacy. It could provide a frequency option for clinical treatment. Furthermore, mechanical research points to a possible connection between the DRG's regulation of P2X3 signaling and the antiallodynic impact of EA. EA can lower nociceptive neural markers (IB4, TRPV1, SP, and CGRP) in the DRG of CCD model rats and proinflammatory cytokines (TNF-α and IL-1β). Our findings imply that EA could be a viable option for CCD management in the clinic.

## Data Availability

Not applicable.

## Abbreviations

EA: Electroacupuncture
CCD: Chronic compression of the dorsal root ganglion
PWT: Paw withdrawal threshold
P2X3: Purinergic receptor P2X3
IB4: Isolectin B4
TRPV1: Transient receptor potential vanilloid
SP: Substance P
CGRP: Calcitonin gene-related peptide
DRG: Dorsal root ganglion
PSN: Persistent spontaneous nociceptive sensation
AUC: Area under the curve
